# Neuromuscular Diseases Care in the Era of COVID-19

**DOI:** 10.3389/fneur.2020.588929

**Published:** 2020-11-26

**Authors:** Bernat Bertran Recasens, Miguel Angel Rubio

**Affiliations:** Neuromuscular Unit, Neurology Department, Hospital del Mar, Barcelona, Spain

**Keywords:** neuromuscular disease (NMD), COVID-19, ALS (amyotrophic lateral sclerosis), CIDP, myasthenia gravis, telemedicine, telehealth

## Abstract

The COVID-19 pandemic has pushed health systems to their limit and forced readjustment of standards of care for different pathologies. Management of neuromuscular diseases becomes a challenge since most of them are chronic, disabling, progressive, and/or require immunosuppressive drugs. There are three main aspects of COVID-19 that affect neuromuscular diseases care. The first one relates to how SARS-CoV2 directly affects different neuromuscular pathologies. Respiratory weakness, as seen in myasthenia gravis, amyotrophic lateral sclerosis, and myopathies, and the use of immunomodulatory drugs (Myasthenia Gravis and Chronic Inflammatory Demyelinating Polyneuropathy) make this group of patients potentially more vulnerable. Secondly, safety measures also affect proper care, limiting care continuity, and physical rehabilitation (one of the essential aspects of myopathies treatment). Telemedicine can partially solve the problem allowing for a continuum of close care, avoiding unnecessary visits, and even guaranteeing the attention of professionals from tertiary care centers. However, one of the crucial steps in neuromuscular diseases is diagnosis, and in most scenarios, more than one face-to-face visit is needed. Lastly, the global COVID-19 situation will also have an economic impact on patients and their families. This situation is of particular concern given that neuromuscular diseases already present difficulties due to the scarcity of resources in terms of public healthcare and research.

## Introduction

The global pandemic situation due to COVID-19 has led to restructuring the management of different diseases. Beyond the transitory periods of total confinement that have occurred in most countries, the current situation entails a restriction of mobility due to the risk of infection of a disease for which, at the time, there is no curative treatment or vaccine. This threat has led to changes in healthcare paradigm: limited access to health centers, reconsideration of the gold standard of excellence in disease management, a thorough evaluation of the risk/benefit balance of specific interventions in the face of drug-disease interactions for a little-known infection and re-thinking of clinical trial procedures to ensure remote participation (from enrollment, dispensing of medication, and follow-up).

Neuromuscular diseases (from now on, NMD) are a heterogeneous group in terms of etiology, genetics, physiopathology, and treatments, that accounts from 2.8 to 18% of the referrals in the neurology department (1). Despite this heterogeneity, many share that they are complex, rare, chronic, disabling diseases, and can follow a progressive course. NMDs often involve the respiratory muscles or are treated with immunosuppressive treatment; which, suppose a group of potential risk for the SARS-CoV2 infection. It is important to emphasize that patients with neuromuscular diseases should not be denied medical care on the basis of stereotypes or assessments of quality of life. Intensive care unit (ICU) doctors should contact the patient's permanent medical practitioner and/or expert center to understand the specific disease history and treatment plan of the patient.

In this paper we will review different aspects where NMDs are affected by this scenario.

## NMD Manifestations of COVID-19 Patients

In many countries, cases of neurological disease, induced by the viral infection itself or by the secondary inflammatory reaction, have been reported. These include stroke, encephalopathy, myelitis, and peripheral nervous system diseases (2). In general terms, SARS-CoV2 is believed to have neurotropism and can access the central nervous system through the olfactory nerves as well as other cranial nerves. This could explain why some patients have persistent anosmia or other cranial neuropathies (3, 4). At the level of the peripheral nerve, the mechanisms that cause its involvement are not fully understood. The nerve may be affected by direct cytotoxic damage from the virus, by systemic inflammation, or by molecular mimicry (4).

Regarding NMD, cases of isolated neuropathies of cranial nerves, acute paralysis similar to Guillain-Barré syndrome (GBS), Miller Fisher syndrome, and increased creatine kinase (CK) have been reported.

On a pathophysiological basis, GBS is considered an immune-mediated disease, but parainfectious cases have been seen during the COVID-19 pandemic. Thus, it is possible that the virus or the inflammation secondary to it damages the nerve (3) However, in most cases, the most widely accepted hypothesis is that a process of molecular mimicry occurs. SARS-CoV2, apart from binding to the angiotensin-converting enzyme 2 (ACE2), binds to glycoproteins and gangliosides on cell surfaces. This last interaction makes it plausible that a cross-reaction occurs between SARS-CoV2 spike-bearing gangliosides and sugar residues of surface peripheral nerve glycolipids (3, 4). The hypothesis of molecular mimicry is supported by the median time interval between COVID-19 symptoms and GBS onset (11.5 days), the acellular cerebrospinal fluid (CSF), and that many of them have responded favorably to IVIG (5, 6). Antiganglioside antibodies are likely not involved because it has only been reported the detection of GD1b antibodies in a patient with Miller Fisher syndrome (7). At the clinical level, the most frequent phenotype of GBS in SARS-CoV2 infection is the classical sensorimotor demyelinating GBS, but all variants and subtypes have been reported. Although one third of GBS patients have respiratory dysfunction, more than 70% have a good prognosis with immunoglobulin treatment (5, 6).

CK elevation, in some cases, appears to be secondary to myositis of probable autoimmune etiology (autoimmune necrotizing myositis: NAM), although direct damage from the virus cannot be ruled out since skeletal muscle cells also express ACE2 (4).

Due to the large number of patients requiring admission to the ICU due to COVID-19 infection, several cases of critical illness myopathy (CIM) (8) as well as compressive neuropathies or plexopathies (many of them associated with the prone position) have been reported. The early recognition of CIM is essential because it allows adaptation to a neuromotor and respiratory rehabilitation plan (9). For its diagnosis neurophysiological studies are crucial, however, sometimes, findings found can be misinterpreted as critical illness polyneuropathy or GBS. As an example, absence of F waves can be found in CIM, but reverses after 1 s burst of 20 Hz repetitive nerve stimulation (10).

Finally, to date, only 3 possible cases of myasthenia gravis debut just after COVID-19 have been described (11).

Thus, since current data seem to indicate that COVID-19 infection can induce not only GBS but also other autoimmune diseases, it is essential to pay attention to different neurological symptoms in those patients.

## COVID-19 Infection in NMD Patients

In the literature and from our own experience, few patients with NMD have presented infection. This could be because they are aware of risks and took preventive measures, but also suggest that they are not at an increased risk of infection or severe illness. Even though certain patients with NMD who require immunosuppressive treatment or ventilatory support have a higher risk of infections, the risk of COVID-19 is not fully known and has been assumed by extrapolation. Thus, it seems logical to think that the poor prognostic factors are the same as the general population (age, obesity, cardiovascular risk factors, chronic high-dose corticosteroid treatment) (12).

There is little data regarding patients with NMD and COVID-19 infection. Cases of patients with myasthenia gravis who have had a SARS-CoV2 infection with a favorable evolution have been reported despite the fact that some have had to change their immunosuppressive treatment or have received treatment with hydroxychloroquine and/or antivirals (13–16).

There are not many reported cases of patients with other neuromuscular diseases such as immune-mediated neuropathies or ALS, and therefore, although always subject to a bias, could indicate that they do not seem to have a more severe infection or higher mortality.

Special caution must be focused in the use of certain therapies for the infection in NMD patients. During the pandemic, due to the need for urgent therapy, drugs that were believed to be effective (e.g., chloroquine or hydroxychloroquine, azithromycin, antivirals, dexamethasone and IL- inhibitors, etc.) have been administered. Actually in the first clinical studies conducted, only remdesivir and dexamethasone are effective (17, 18). In contrast, both hydroxychloroquine and azithromycin appear to be ineffective (19).

Azithromycin and chloroquine should be avoided, specifically those with MG, as both have been associated with a worsening of the disease (20). Since hydroxychloroquine additionally supposes a risk for skeletal and cardiac muscle because with life-threatening cardiac arrhythmias is important to avoid in patients with NMD and cardiomyopathy as in DMD/BMD.

Regarding remdesivir, although some protease inhibitors had been associated with polyneuropathy, due to the short duration of treatment, only a few cases of toxic myopathy have been reported. In the case of MG, there is no evidence that remdesivir can induce clinical worsening (21).

Patients with COVID-19 have also an increased risk of thromboembolic events and therefore anticoagulant prophylaxis should be considered in patients that require also IVIG due to their underlying disease.

Finally, respiratory management has to be similar to the general population, taking into account the greater risk of respiratory muscles dysfunction.

## Impact of COVID-19 Pandemic in the Care of NMD Patients

### Diagnosis

One common problem of many NMD is the diagnosis delay and electrodiagnostic testing (EDX) plays an essential role. The restricted access to health centers has also limited the performance of EDX studies, as they require close contact with the patient over a prolonged period, and therefore could lead to further delay in diagnosis. Priority measures to ensure adequate safety in the studies are the use of personal protective equipment (PPE) and N95 masks, and the use of checklist of symptoms the day before the study. In cases of high-risk studies (i.e., facial studies, laryngeal electromyography), it is recommended to request polymerase chain reaction (PCR) studies on nasopharyngeal samples 24–48 h in advance (22). Follow-up EDX exams that are not urgent, do not contribute to a change in management and pose an increased risk of infection, can be postponed.

### Treatment

Immunosuppressive treatment (including thymectomy in myasthenia gravis—MG) increases the risk of infections in patients with MG, Chronic Inflammatory Demyelinating Polyneuropathy (CIDP), or inflammatory myopathies. MG patients had an increased risk of infection of 39% (23), with pneumonia (especially bacterial) as the most frequent type of infection followed by sepsis, cutaneous, and soft tissue infections (24).

In the particular case of COVID-19, as mentioned above, there is little data of real risk of infection in NMD patients. However, several cohorts of patients with autoimmune diseases such as psoriasis, rheumatoid arthritis, multiple sclerosis, or optic neuromyelitis show that treatment with immunosuppressants does not increase the risk of suffering from COVID-19 and neither present a more severe form of infection (25–28).

However, since current data corresponds to a small number of patients, it seems reasonable to continue with the indications of the expert groups; individualize decisions regarding the patient's situation or comorbidities, modify the immunosuppressive drug to one with a better safety profile (i.e., intravenous immunoglobulins, plasmapheresis, or steroids), space out the doses or even postpone the initiation of the medication in cases of stable patients or those with mild symptoms (29).

Long term corticosteroids is the drug that increase most the risk of infections (20–50%) (23, 24) and are often administered in various NMDs: MG, chronic dysimmune neuropathies, Duchenne/Becker Muscular Dystrophy (DMD/BMD) and patients with spinal muscular atrophy (SMA) treated with Onasemnogene abeparvovec-xioi (steroids must be administered for the first 2 months since the administration of the gene therapy). Although considered a risk factor given the associated immunosuppression, in general terms they should not be discontinued abruptly as they can cause a worsening of the underlying disease.

Plasmapheresis and intravenous immunoglobulins (IVIG) do not appear to increase the risk of infections, probably because they are often given in short courses, although long-term effect is unknown (30). However, a balance must always be made between the risk and benefit of each situation. For example, in CIDP patients stable for at least 6 months and treated with IVIG, the need for a new dose of IVIG could be reappraised (29, 30). Also, although plasmapheresis is not routinely used in these patients and is usually reserved for refractory cases, an US expert panel considers the inclusion of plasmapheresis as a second option over corticosteroids (31).

Prada et al., reported a cohort of 196 patients affected by immune-mediated neuromuscular diseases, with 0.6% laboratory-confirmed COVID-19 infection in those receiving IVIG or subcutaneous immunoglobulins, and 6.4% confirmed infection in those with other immunosuppressive treatment or no treatment. This could suggest a protective effect of chronic immunoglobulin therapy in the risk of COVID-19 infection. However, more studies are warranted to confirm this (32).

In patients with DMD/BMD, it is recommended to maintain the current treatment, including steroid therapy and oligonucleotide treatments (33). There have been concerns about the use of ACE inhibitors given the role of ACE2 in SARS-CoV2 infection. Numerous scientific societies such as the American Heart Association have stated that ACE inhibitors should not be withdrawn from patients as they have shown benefits in different cardiac conditions and a detrimental effect on coronavirus infection has not been yet demonstrated. Henceforth, patients with DMD/BMD who take such drugs should continue to take them (33).

Nowadays, several pharmacological treatments improve survival and motor skills of SMA patients. These treatments are nusinersen and gene therapy with Onasemnogene abeparvovec-xioi. Both have improved the quality of life, motor function and increased survival; and although these treatments are new, they should never be considered as elective or expendable. The delay of their administration should be avoided whenever possible, assessing the risks on an individual basis. In the case of nusinersen (intrathecal administration with a loading dose of 4 doses the first 2 months and then every 4 months), it has been reported that the delay of 1 month without medication leads to a 10% reduction of the drug in CSF. If there is a delay in administration, the schedule should not be readjusted, but rather follow initial proposed calendar to ensure optimal intrathecal medication levels (34).

Regarding other immunomodulatory treatments (mycophenolate mofetil, cyclophosphamide, azathioprine, etc.), the increased risk of infection is similar to corticosteroids (35). Also, when the risk-benefit ratio of each case is considered, given the possibility of exposure to infection, it should be kept in mind that some of these drugs have no proved efficacy in some NMDs (i.e., rituximab, azathioprine, methotrexate, or mycophenolate in CIDP).

Due to the fact that patients with NMD present frequent respiratory infections, it is important to do a close follow-up (by professionals of the multidisciplinary Unit as well as from primary care). As for household measures, ventilation devices, unless equipped with bacterial/viral filters (HEPA), as well as airway clearance devices (cough assist, nebulization), increase the risk of particle dispersion into the environment. Despite this, patients and caregivers should be warned not to modify the ventilation schedule to avoid errors. If possible, it is recommended to convert the tubing and mask circuitry to a closed system by using both a double-lumen tube with a viral/bacterial filter and a non-vented full-face mask to restrict viral spread (36).

During confinement and, currently, due to social distancing measures, the population is doing less physical activity (37). This change most significantly affects patients with NMD. That is why, rehabilitation continuity must also be guaranteed, either face-to-face or by telematics communication.

A brief summary of the considerations to be taken into account in the management of the different NMDs is shown in Table 1.

**Table 1:** Neuromuscular disorders management in Covid-19 era.

- Encourage telematics assessments ° Monitoring home ventilation ° Perform respiratory assessments if able - Do not stop rehabilitation - Psychological support (specially in DMD with behavioral disorders) - Perform EDX safely and DO NOT delay diagnosis - Consider clinical trial monitoring remotely - Patients under steroids treatment: do not stop steroids and consider increasing the dose in ill patients - Rationalize the use of IS agents ° Space doses or postpone the initiation if stable (especially if the infusion is in the hospital: cyclophosphamide, rituximab, etc.) ° Choose IS with a safer profile. Do not stop IACE drugs ° Consider switching IVIG to subcutaneous immunoglobulins ° Consider PLEX or IG as an alternative option instead steroids in CIDP patients ° For maintenance rituximab therapy, consider delaying the infusion ° even beyond 6 months, if the CD19 and CD20 lymphocytes are suppressed - Consider anticoagulant prophylaxis in patients with COVID-19 that require also IVIG - Continue ASO treatment as scheduled in SMA patients - Do not stop IACE drugs (DMD/BMD) - Avoid chloroquine, hydroxychloroquine (DMD/BMD and MG) and azithromycin (MG)

*Summary of recommendations for the management of neuromuscular diseases. MG, myasthenia gravis; DMD/BMD, Duchenne/Becker muscular dystrophy; SMA, spinal muscular atrophy; ALS, amyotrophic lateral sclerosis, IS, immunosuppressants; IG, immunoglobulin; IVIG, intravenous immunoglobulin; PLEX, plasmapheresis; IACE, angiotensin-converting enzyme inhibitors; ASO, antisense oligonucleotide*.

### Clinical Trials

The status of clinical trials in NMD has been impaired during the pandemic situation with difficulties with new inclusions, treatment supplies and monitoring. Data reported from the NEALS (38) on the status of ALS clinical trials during the pandemic show that very few sites were able to recruit new patients and that 20% of follow-up patients withdrew from the trial. This should make us consider new approaches to clinical trials.

Thus, in a situation in which the recruitment of patients with rare diseases is already complicated (39), several strategies must be considered. Some examples are: advising the population about studies on the Internet or social network advertisements; the use of telematic consent (tele-consent) or the utilization of telephone interviews; mobile applications or other devices such as dynamometers, actigraphs, accelerometers, or remote measurements of respiratory function. Also, the need for remote dispensing and collection of medication should be addressed.

Telematic assessment of progression could evaluate more frequently and in daily environment for the patient and in turn could even reduce the duration and the number of subjects needed in clinical trials.

Finally, it is essential to prevent selection bias toward patients with ease in remote monitoring and more familiar with technology.

### Socioeconomical Impact

One of many faces of catastrophic COVID-19 situation is its social, occupational and economic impact in NMD patients and their families. For example, the estimation of the annual cost of an ALS patient is approximately 50,000 euros (1), and 70,000 euros for SMA patients, being even higher in SMA type 1 (107,000 euros/year) (40). Since their essential care can be directly affected by economic situation, NMDs are at risk, in terms of personal healthcare, in a context of deterioration of the individual and global economy.

Other consequences are the increase in anxiety and feelings of loneliness that have been reported in NMD patients and their relatives/caregivers during confinement (41). In the case of ALS patients have been described worsening of depressive and anxiety symptoms, associated to altered interoceptive awareness during the outbreak (42).

It is essential not to forget the psychological support for the patient, his family and the caregivers, even if it is remotely. As an example, DMD/BMD boys may have a spectrum of behavioral disorders, including some degree of intellectual disability and anxiety problems which will require close psychological follow-up.

Finally, because research is currently focused on finding therapeutic strategies for COVID-19, there is a redirection of interest (and thus funding) which may lead to a loss of already fragile research funding in NMD.

### Ethical Issues Related to NMD Patients Care

In situations with a high healthcare burden, it is important not to discriminate patients with chronic and rare diseases, from a therapeutic point of view. ICU doctors should discuss the case with the patient's permanent medical practitioner and/or expert center to understand the disease history and treatment plan of the patient (43).

## Future Perspectives

### Vaccines

Multiple clinical trials are currently underway to develop an effective vaccine against COVID-19. There are more than 120 vaccine candidates but neither uses live attenuated virus (44), and therefore those under immunosuppressive treatment could receive it. However, it is not known whether immunomodulatory treatment can alter the efficacy of vaccines and hence, what would be the best time to vaccinate according to the immunosuppressive treatment scheme (45). Therefore, when the occasion arrives, it will be crucial for the patient to coordinate with his/her referring neurologist.

### Beyond the Hospital Walls. Tele-Health in Times of COVID-19

#### Remote Care and Management

The current situation has pushed the limits of telehealth in NMDs, both in terms of implementation in medical centers and in terms of acceptability and skepticism by patients and health professionals. The gold standard of healthcare is multidisciplinary consultation, but this often involves drawbacks since patients and their families might spend many hours traveling and staying at the hospital with the fatigue and burden that this involves. Moreover, as the disease progresses and the problems of mobility and fatigue increase it becomes more challenging to travel and, conversely, contact with medical services becomes more necessary. Tele-health services must be conceived as an additional complimentary activity to face-to-face visits. Therefore, remote assistance is an essential opportunity for NMDs, even more so in this current pandemic situation. A survey of NEALS (North East ALS Consortium) to know the impact of the COVID-19 situation on ALS patient's healthcare and research (38) revealed that one third of the centers had not been able to visit new patients in person, more than half had visited via video-call, and only 16% of the centers were able to fully supply their patients with the basics (gastrostomy tubes, wheelchairs, medication, etc.).

In addition, the telehealth model allows the patients to hold the consultation in familiar environments, making it more comfortable to them, maintaining the link with multidisciplinary units even in more advanced stages (46).

Nowadays, we know that specific functional scales or aspects of physical examination can be performed telematically with strong correlation with their equivalents in face-to-face visits performed by health professionals (47). These methods not only can replace 'traditional' face-to-face visit but they can also be used to keep closer control of the evolution of the patients and even be essential for therapeutic decisions, such in the case of chronic dysimmune neuropathies. For this reason, it is crucial to be able to perform objective and validated measures remotely. This monitoring can be carried out through telephone interviews, questionnaires in mobile apps or even through 'user-friendly' devices, adaptation of functional scales (Inflammatory Neuropathy Cause and Treatment—INCAT disability score, Inflammatory Rasch-built Overall Disability Scale—I-RODS, ALSFR-R), by using dynamometers, through remote physical examination or by asking about activities of daily living, fatigue and falls. It is easy to imagine that a video call allows, for example, to observe the lifting capacity of the arms or the wandering. Although there may be concerns about the validity of these measurements, it should be noted that the Medical Research Council (MRC) scale also has inter- and intra-observer variability.

Respiratory function monitoring is also essential since provide prognosis information, indicates proper timing of non-invasive ventilation, and are a crucial key of inclusion criteria of most clinical trials. However, there is a risk of producing aerosols during the examination. There is evidence of viability of home respiratory screening devices with a reasonable degree of correlation with in-hospital respiratory screening and hence its use must be encouraged. Several NMD finally will require the use of home mechanical ventilation. In many cases, it is possible to control the parameters from the hospital, avoiding unnecessary displacements.

## Discussion

Living with a NMD is a challenge not only for patients but for their caregivers and families, and COVID-19 pandemic situation represents even a greater stress test.

NMD patients are a group at risk, either because of respiratory affectation or their condition of immunosuppression. For this reason, it is important to individualize the management of NMD patients, considering the interventions and treatments individually and taking into account the risk of going to the hospital and the risk of not receiving proper attention.

On the other hand, telemedicine has suffered an accelerated expansion, representing an opportunity to improve clinical care management and research. Ideally, a combination of telematics and face-to-face visits would create a hybrid model that would be defined depending on the evolutionary moment of each patient.

The gold standard in the management of NMDs is the attention in multidisciplinary units, where holistic and global management of the patient's needs is given, as well as research and clinical trials are performed. Multidisciplinary units must continue to commit to excellence in care, but now in a remote scenario in which the wellness of the patient must still be the focus even in these uncertain times.

## Data Availability Statement

The original contributions presented in the study are included in the article/supplementary material, further inquiries can be directed to the corresponding author/s.

## Author Contributions

All authors contributed to the study conception and design. The first draft of the manuscript was written by MR and BB commented on previous versions of the manuscript. All authors read and approved the final manuscript.

## Conflict of Interest

The authors declare that the research was conducted in the absence of any commercial or financial relationships that could be construed as a potential conflict of interest.
